# Anatomy relevant to cholecystectomy

**DOI:** 10.4103/0972-9941.16527

**Published:** 2005-06

**Authors:** Sanjay Nagral

**Affiliations:** Department of GI Surgery, Jaslok Hospital and Research Centre, G Deshmukh Marg, Mumbai, India

**Keywords:** Anatomy, Biliary, Cholecystectomy, Gallbladder, Injury, Laparoscopy

## Abstract

This review discusses anatomical facts that are of relevance to the performance of a safe cholecystectomy. Misinterpretation of normal anatomy and anatomical variations contribute to the occurrence of major postoperative complications like biliary injuries following a cholecystectomy, the incidence being higher with laparoscopic cholecystectomy. A look at the basic anatomy is therefore important for biliary and minimally invasive surgeons. This includes normal anatomy and variations of the biliary apparatus as well as the arterial supply to the gallbladder. Specific anatomical distortions due to the laparoscopic technique, their contribution in producing injury and a preventive strategy based on this understanding are discussed. Investigative modalities that may help in assessing anatomy are considered. Newer insights into the role of anatomic illusions as well as the role of a system-based approach to preventing injuries is also discussed.

## INTRODUCTION

Knowledge of relevant anatomy is important for the safe execution of any operative procedure. Specifically, in the context of a cholecystectomy, it has been recognized since long that misinterpretation of normal anatomy as well as the presence of anatomical variations contribute to the occurrence of major postoperative complications especially biliary injuries.[1] Such injuries in turn can cause significant morbidity and occasionally even mortality. They are also one of the commonest causes of litigation against abdominal surgeons in the developed world. There is now a fair amount of data to suggest that the acceptance of laparoscopic cholecystectomy (LC) as the standard procedure, has led to an increase in bile duct injuries.[2] This seems partly related to the different anatomical exposure of the area around the gallbladder especially the Calot's triangle during the laparoscopic procedure as opposed to the open procedure.

Hence, it is important for biliary and minimally invasive surgeons to appreciate basic anatomical facts as they apply to the performance of cholecystectomy as well as understand from literature how anatomical distortions or variations can contribute to complications. This review attempts to address these issues. It is not an exhaustive description of biliary anatomy but discusses anatomical facts that are of relevance to the performance of a safe cholecystectomy.

## BASIC ANATOMY

### Gallbladder

The gallbladder is a pear shaped organ situated in a fossa on the liver undersurface. It is variable in shape and volume. Normally present at the junction of segments 4 and 5 (and at the lower limit of the principal plane or Cantlie's line) its position in relation to the liver may vary. For example, it may be partially or completely embedded within the liver parenchyma, the so-called ‘intrahepatic’ gallbladder. This may create difficulties in dissection and may increase the chance of intraoperative injury to the liver. Although the main right pedicle is fairly deep in the liver parenchyma, large portal, and hepatic venous branches traverse the liver at a depth of around one cm from the gallbladder. Thus, a deep liver tear during the dissection of the gallbladder off its fossa can occasionally bleed profusely. Also, during the dissection it may be important to err on the side of the gallbladder rather than the liver parenchyma.

The gallbladder is divided into a fundus, a body and a neck or infundibulum. The ‘Hartmann's pouch’ an out pouching of the wall in the region of the neck is recognized more as an outcome of pathology in the form of dilatation or presence of stones.[3] This pouch is variable in size but a large Hartmann's pouch may obscure the cystic duct and the Calot's triangle. This may be result of plain enlargement or due to adherence to the cystic duct or bile duct. Thus a small cystic duct can get completely hidden and traction on the gallbladder can lead to the bile duct looking like the cystic duct. An exaggerated form of the same process is the ‘Mirizzi's syndrome’ in which a large stone in the Hartmann's pouch area is either adherent to or erodes into the bile duct. This can create major difficulty during a cholecystectomy.

Although the accessory ducts are discussed separately later in the article the cholecysto-hepatic duct can join the gallbladder at any point in its hepatic bed. The exact incidence of such ducts is not well documented and in fact some authors question their existence.[3] Thus, a duct encountered in the gallbladder fossa is likely to be a small superficial intrahepatic duct and can be ligated safely.

### Cystic duct

The cystic duct joins the gallbladder to the bile duct and is one of the important structures needing proper identification and division during a standard cholecystectomy. The cystic duct may run a straight or a fairly convoluted course. Its length is variable and usually ranges from 2 to 4 cm.[3] Around 20% of cystic ducts are less than 2 cm. Hence there may be very little space to put clips or ligatures. True absence of the cystic duct is extremely rare[3] and if the duct is not seen is more likely to be hidden. The cystic duct is usually 2–3 mm wide. It can dilate in the presence of pathology (stones or passed stones). The normal bile duct is also around 5 mm and hence can look like a mildly dilated cystic duct. In general a cystic duct larger than 5 mm (or the need to use a very large clip to completely occlude the duct) should arouse a suspicion of mistaken identity with the bile duct before it is clipped or ligated.

**Figure 1:**
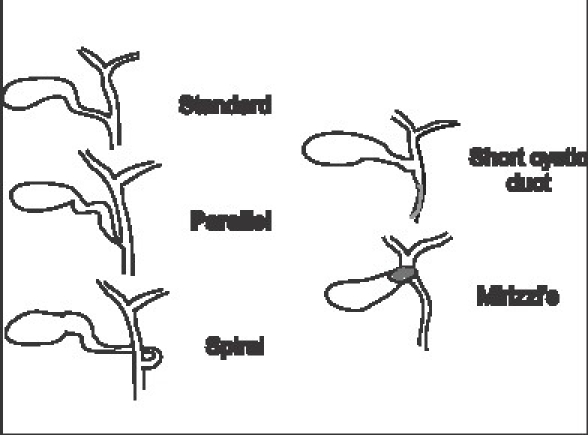
Modes of union of cystic duct with bile duct

The cystic duct joins the gallbladder at the neck and this angle may be fairly acute. Also the mode of joining may be smooth tapering or abrupt. On the bile duct side its mode of union shows significant variations [Figure 1]. Since such variations are not uncommon it may not be safe to try and dissect the cystic duct to its junction with the bile duct. It is important to remember that even in the low insertion variety the cystic duct rarely goes behind duodenum and therefore a ductal structure passing behind the duodenum is more likely to be the bile duct itself. Double cystic ducts are described but are exceedingly rare and therefore two ductal structures entering the gallbladder should always be viewed with suspicion. Also the cystic duct does not have vessels traveling on its surface whereas the bile duct has such visible vessels.[2]

### Cystic artery and right hepatic artery

The cystic artery is a branch of the right hepatic artery (RHA) and is usually given off in the Calot's triangle. It has a variable length and enters the gallbladder in the neck or body area. The course and length of the cystic artery in the Calot's triangle is variable. Although classically the artery traverses the triangle almost in its center, it can occasionally be very close or even lower than the cystic duct.

**Figure 2:**
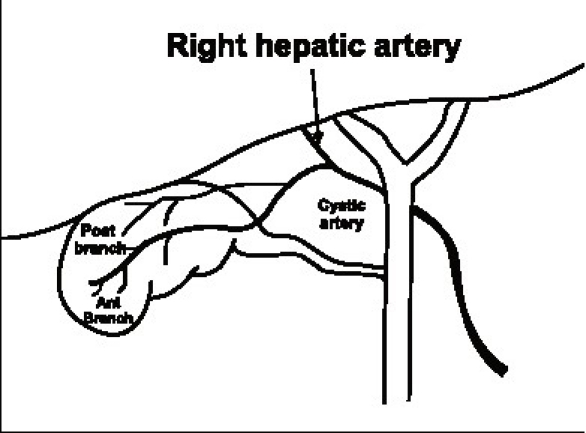
Anterior and posterior branches of the cystic artery

It usually gives off an anterior or superficial branch and a posterior or deep branch. This branching usually takes place near the gallbladder. When the point of dissection is very close to the gallbladder as in a LC or the branching is proximal, one may have to separately ligate the two branches [Figure 2]. Also if the presence of a posterior branch is not appreciated it can cause troublesome bleeding during posterior dissection. In addition the cystic artery gives of direct branches to the cystic duct. These small vessels have been better appreciated in the era of LC and need to be divided to obtain a length of cystic duct before division.

**Figure 3:**
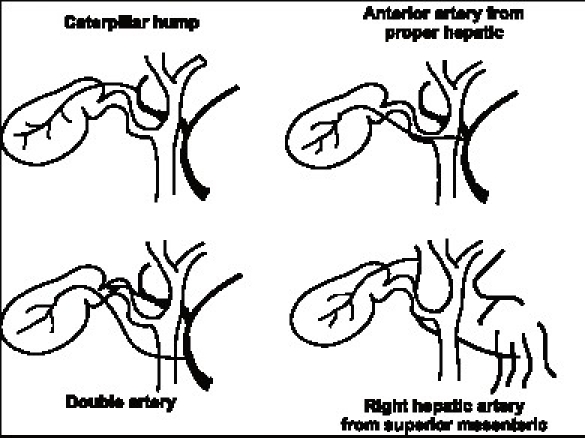
Some variations of the arterial supply to the gallbladder

The RHA normally courses behind the bile duct and joins the right pedicle high up in the Calot's triangle. It may come very close to the gallbladder and the cystic duct in the form of the ‘caterpillar’ or ‘Moynihan's’ hump [Figure 3]. Although the incidence of this variation is variable it seems common enough to merit detailed description and may be as high as 50%.[3] If such a hump is present, the cystic artery in turn is very short. In this situation the RHA is either liable to be mistakenly identified as the cystic artery or torn in attempts to ligate the cystic artery. The ensuing bleeding in turn predisposes to biliary injury.

There are a fair number of other arterial variations of the cystic artery also described [Figure 3]. Many of these are unlikely to cause confusion if the artery is divided very close to the gallbladder wall. There is a 2–15% incidence of double cystic artery. Therefore it may be occasionally necessary to ligate two arteries to the gallbladder. When the cystic artery is given off not from the RHA but from other vessels like the common hepatic artery or the left hepatic artery (2–5%) it crosses the bile duct anteriorly and may be prone to injury. Also the superior mesenteric artery may give off the cystic artery in which case it ascends to the gallbladder below the cystic duct. An accessory or replaced RHA from superior mesenteric artery which is a variation seen in almost 15% of individuals the RHA courses thru the Calot's triangle (and therefore nearer the gallbladder) and in turn has a shorter cystic artery.

### Accessory and aberrant ducts

**Figure 4:**
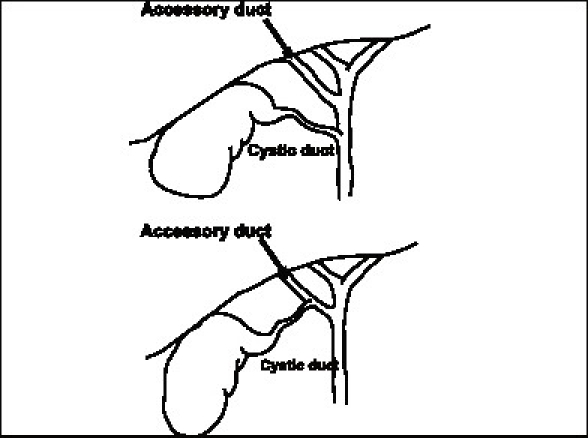
Accessory bile ducts relevant to cholecystectomy

There are a large number of accessory ducts described in the biliary drainage network of the liver. However, the accessory ducts likely to be encountered during a cholecystectomy are those draining parts of the right lobe. These ducts are typically small and course through the Calot's triangle (and therefore closer to the gallbladder) before they enter the common hepatic duct separately below the confluence of the right and left duct at variable distances. Sometimes the cystic duct may actually join the accessory duct. Some of the variations of relevance to cholecystectomy are shown [Figure 4].

These ducts may drain substantial portions of the right lobe of the liver, either one of the sectors (two segments) or a segment and may in fact be the sole drainage of that part of the liver in which case they are more precisely termed as ‘aberrant’ ducts. It has been suggested that most such ducts are aberrant rather than accessory[3] in which case it is even more important to safeguard them. Cholangiographic studies have shown that there is almost a 20% incidence of the right anterior or the right posterior ducts joining the common hepatic duct separately rather than in the form of a right duct. If such a duct is injured it can lead to substantial biliary stasis or leak. The size of the duct may be an indirect indicator of the amount of liver it drains. It has hence been recommended that in case of injury if the duct is more than 3 mm it should always be drained into a Roux loop.[3] Alternatively one can perform a cholangiogram through the duct to assess the amount of liver it drains as well as whether it is accessory or aberrant. With increasing recognition of injury to such ducts these have now been grouped into separate type in the recent Strasberg classification[2] of bile duct injuries.

### Calot's triangle

**Figure 5:**
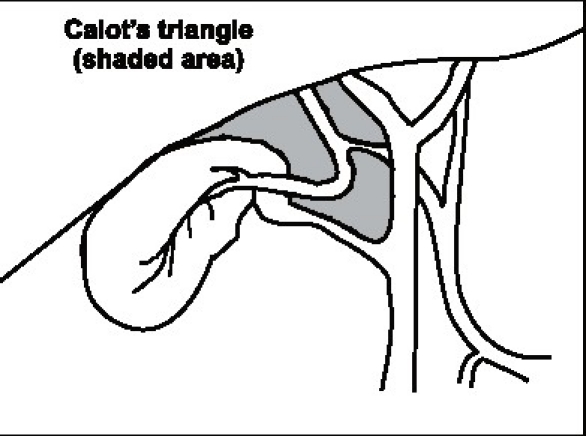
Calot's triangle

This famous triangle was described as bound by the cystic duct, the bile duct and the cystic artery in its original description by Calot in 1891. In its present interpretation the upper border is formed by the inferior surface of the liver with the other two boundaries being the cystic duct and the bile duct [Figure 5]. Its contents usually include the RHA, the cystic artery, the cystic lymph node (of Lund), connective tissue, and lymphatics. Occasionally it may contain accessory hepatic ducts and arteries as discussed previously. It is this triangular space, which is dissected in a cholecystectomy to identify the cystic artery and cystic duct before ligation and division. In reality, it may be a small potential space rather than a large triangle making the dissection of its contents without damaging the bordering structures the most challenging step of a cholecystectomy. In addition the space may be obscured and shrunken by various mechanisms. The left (or medial) boundary of the triangle formed by the bile duct is the most important structure, which needs to be safeguarded.

### Laparoscopic anatomy

**Figure 6:**
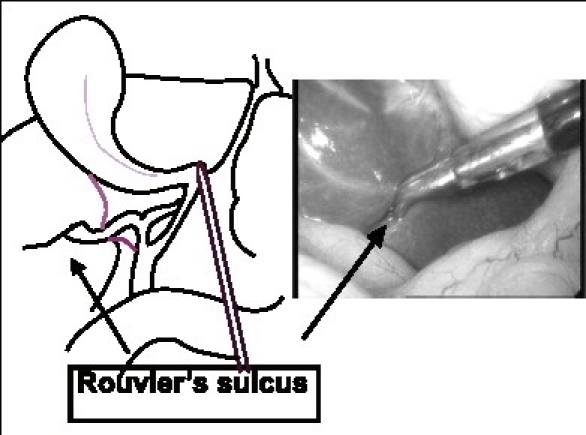
Rouviere's sulcus

**Figure 7:**
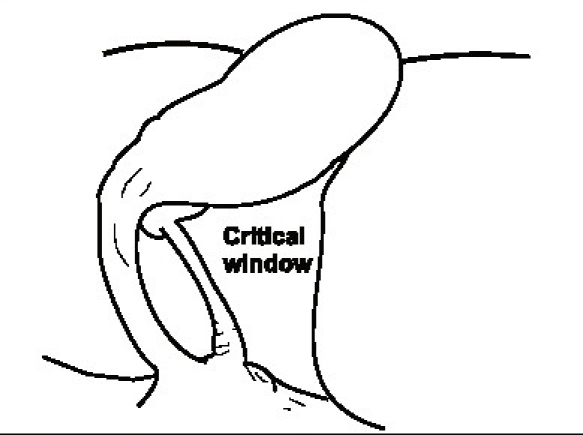
The critical view or safety window

The advent and popularity of LC has led to a new look and insights into biliary anatomy especially of the Calot's triangle area and the term ‘laparoscopic anatomy’ has actually found a place even in anatomy texts. Although a detailed discussion of all the factors peculiar to laparoscopy that contribute to an increased incidence of injuries is beyond the purview of this review, the different anatomical ‘laparoscopic view’ of the area around the gallbladder especially the Calot's triangle does contribute to misidentification of structures. The method of retraction during the laparoscopic procedure tends to distort the Calot's triangle by actually flattening it rather than opening it out.[2] Also, the reluctance to (or difficulty in) performing a fundus first cholecystectomy during the laparoscopic procedure as opposed to the open procedure also contributes to the same lack of exposure of the Calot's triangle. Finally, the ‘posterior’ or ‘reverse’ dissection of the Calot's triangle, which is popular during an LC, again gives a different view of the area and since the gallbladder is flipped over during this method may lead to further anatomical distortion. The Rouviere's sulcus is a fissure on the liver between the right lobe and caudate process and is clearly seen during a LC during the posterior dissection in a majority of patients[4] [Figure 6]. It corresponds to the level of the porta hepatis where the right pedicle enters the liver. It has hence been recommended that all dissection be kept to a level above (or anterior) to this sulcus[4] to avoid injury to the bile duct. Also, this being an ‘extrabiliary’ reference point it does not get affected by distortion due to pathology. Similarly, a clear delineation of the junction of the cystic duct with the gallbladder along with the demonstration of a space between the gallbladder and the liver clear of any other structure other than the cystic artery (safety window or critical view) is also recommended as an essential step to prevent bile duct injury[2] [Figure 7].

### Investigations to assess the anatomy

Drawings of the Calot's triangle from anatomy texts are very different from the anatomy seen during the performance of a cholecystectomy. In the first place all the structures forming the boundaries of the Calot's triangle are not seen during surgery as they are covered with tissue. Also, in a significant number of individuals since the cholecystectomy is performed for pathology in the form of cholecystitis the anatomy is obscured by inflammation, edema, adhesions, fibrosis, and presence of stones.

In view of the importance of anatomy and it's variations in injuries caused during cholecystectomy it is logical to look at the possibility of assessing the anatomy accurately with the help of imaging before or during the performance of a cholecystectomy.

Most cholecystectomies are performed after identification of gallstone disease on ultrasound examination. Although on occasion an ultrasound examination can predict gross distortions of anatomy like the Mirizzi syndrome, in the usual case it does not throw any light on anatomical relations. Thus knowledge of the specific anatomy in that individual is not available to the surgeon preoperatively as a routine. If a cholangiogram in the form of a magnetic resonance cholangio pancreatography (MRCP) or an endoscopic retrograde cholangiopancreatography (ERCP) has been performed for some reason, it may reveal anomalies like the presence of accessory ducts or a low insertion of cystic duct.

Methods to assess anatomy during the surgery are perhaps more relevant. The first and foremost (and perhaps the most reliable) is clean dissection and accurate visual identification of the contents of the Calot's triangle especially the cystic artery and duct. The role of a routine intraoperative cholangiogram in delineating biliary anatomy and in turn preventing misidentification has been a subject of a long and intense debate amongst biliary surgeons but there is conflicting evidence on its value.[2] In reality most biliary surgeons do not perform a routine intraoperative cholangiogram but use it selectively. In any case, unless it is performed through the gallbladder, once a duct has been opened for a cholangiogram in case it is the bile duct this actually constitutes a partial injury. Also a cholangiogram may not delineate all aberrant ducts and does not provide any insight into arterial anatomy.

Recently, there have been sporadic reports of the use of newer sophisticated technology to identify biliary as well as arterial anatomy during the performance of a cholecystectomy. This has included the use of laparoscopic ultrasound for identification of structures, laparoscopic Doppler for identification of arteries and the use of an instrument called the tactile sensor probe. Some recent reports describe innovative methods such as the injection of a dye called methelenum coeruleum into the gallbladder which gives a blue color to the biliary system and the introduction of a small optical fiber thru ampulla of vater which illuminates the entire biliary tree during the cholecystectomy a procedure called ‘light cholangiography.’[5] Most of these methods rely on costly technology, are largely unavailable and have not been scientifically validated. Thus, it seems that presently there is no good alternative to meticulous dissection in a planned manner with precise identification of structures before they are divided.

Finally, an interesting recent study has shown that ‘anatomic illusions’ to which everyone is susceptible are the primary cause of bile duct injuries; experience, knowledge, and technical skill by themselves may not be adequate protection against such illusions and the resultant complications.[6] The study also suggests that the current incidence of bile duct injury may be nearing the upper limits of human performance and that the most useful corrective strategy may lie outside the individual in changes in the processes or technology. Another similar study recommends that surgeons performing cholecystectomies should have an intraoperative protocol that is similar to navigation principles used in the aviation and maritime industry.[7]

The number of cholecystectomies, especially LCs, being performed in India has increased phenomenally in the last few years. Although there is no large population-based data there is some evidence that the incidence of biliary injuries is increasing as referral units including ours are treating an increasing number of patients every year. While there has been a lot of focus on technology and technical skills, discussions on anatomy and it's relevance in prevention of injuries also deserve space in the future.
